# Growing Teratoma Syndrome: A Rare Outcome

**DOI:** 10.1155/2021/8884787

**Published:** 2021-01-02

**Authors:** João Rebelo, Francisco Moreira, Margarida Morgado, Ana Sofia Preto, António Madureira

**Affiliations:** ^1^Radiology Department, Centro Hospitalar Universitário de São João, Porto, Portugal; ^2^Pathology Department, Centro Hospitalar Universitário de São João, Porto, Portugal; ^3^Faculdade de Medicina da Universidade do Porto, Porto, Portugal

## Abstract

Growing teratoma syndrome is a rare condition described in both testicular and ovarian cancer. We present a case of a 26-year-old male with known mixed germ cell tumor which exhibited new and progressive secondary lesions during imaging surveillance, later to be histologically characterized as teratomas.

## 1. Introduction

Growing teratoma syndrome (GTS) was first reported by Logothetis in 1982, as a rare entity among patients with nonseminomatous germ cell tumors, characterized by growing masses developing during or after chemotherapy despite normal levels of tumoral biomarkers.

A close imaging surveillance is essential for early recognition of GTS, particularly in patients with risk factors, and because of the chemoresistant nature of GTS, there is a need to prevent further uneffective systemic therapy. Surgical resection is currently the gold standard for GTS treatment.

Although GTS typically has a good prognosis, regular follow-up is critical to detect malignant degeneration.

## 2. Case Report

A 26-year-old male was referred to our institution to study a potential mass of the right testicle, noted in the previous weeks on physical exam. The patient had no significant medical background or history of previous testicular trauma. A testicular ultrasound and serum tumoral biomarkers were ordered.

**Figure 1:**
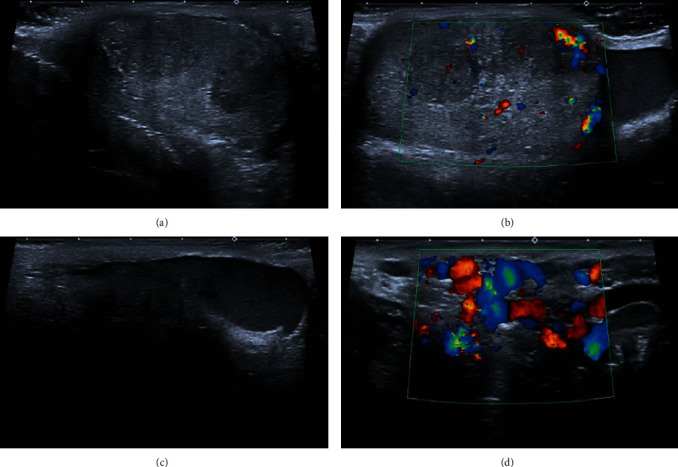
Ultrasound. (a–c) Enlarged right testicle (estimated volume of 32 cc) and a highly heterogenous parenchyma with multiple nodules, most of them hypoechogenic and demonstrating high vascularization. A right complex hydrocele, suggestive of an hematocele, is also seen. (d) Doppler analysis found bilateral varicocele without significant reflux with Valsalva maneuvers.

A testicular ultrasound was performed which showed an enlarged right testicle (estimated volume of 32 cc), presenting highly heterogenous parenchyma with multiple nodules, most of them hypoechogenic and demonstrating high vascularization. A right moderate hematocele was also present. Doppler analysis also showed bilateral varicocele (pampiniform plexus venous caliber above 3 mm), without significant reflux with Valsalva maneuvers. This finding precluded an abdominal ultrasound to exclude extrinsic causes for venous congestion. It revealed one superior retroperitoneal lymphadenomegaly with almost 4 cm on its major axis, associated with densification of its surrounding fat. Ultrasound findings suggested a testicular mass suspicious for malignancy with metastatic retroperitoneal lymph node (Figure 1).

**Figure 2:**
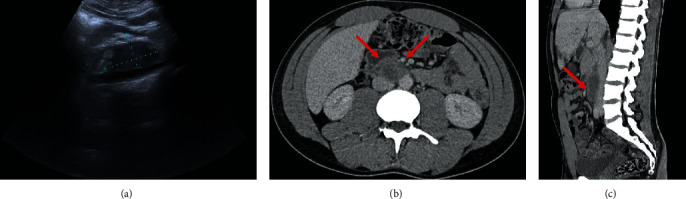
Ultrasound and CT. (a) Sagittal gray-scale abdominal ultrasound of the superior retroperitoneum, demonstrating a hypoechoic enlarged lymph node (between calipers). (b, c) Axial and sagittal contrast-enhanced CT abdominopelvic images showing the same enlarged lymph node (red arrows), on staging. Note the discrete fat stranding in the adjacent planes.

**Figure 3:**
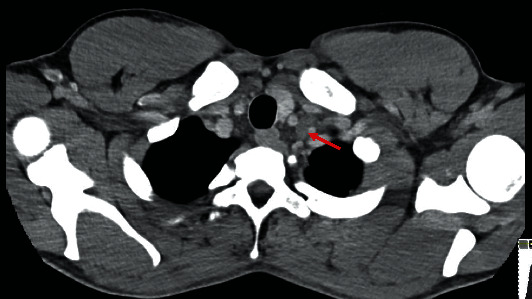
CT. Axial thoracic contrast-enhanced CT image at the thoracic inlet, showing a left supraclavicular lymphadenomegaly (red arrow).

A thoracoabdominopelvic computed tomography (CT) with IV contrast was performed for staging, confirming a retroperitoneal lateroaortic lymphadenomegaly and also finding a left supraclavicular lymphadenomegaly (2 cm of major axis). No other signs of secondary lesions were found (Figures 2 and 3).

**Figure 4:**
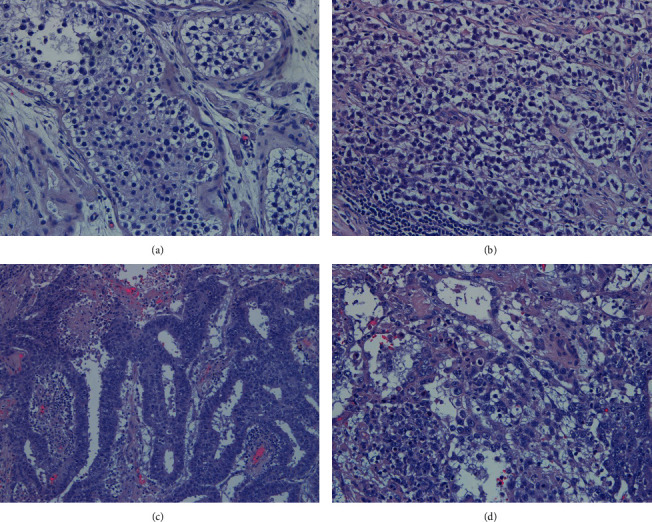
Testicular involvement by mixed germ cell neoplasm associated with germ cell neoplasia in situ (a, H&E 200x), with seminoma-type components (b, H&E 200x), embryonal carcinoma type (c, H&E 200x), and yolk sac type (d, H&E 200x).

**Figure 5:**
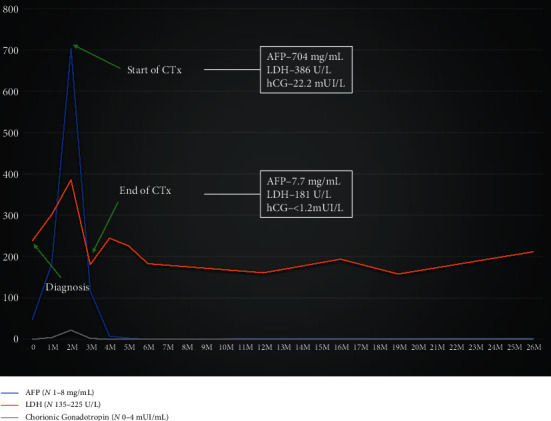
Evolution of tumoral biomarkers serum levels throughout patient follow-up, since diagnosis to the present day. AFP: alpha fetoprotein; LDH: lactate dehydrogenase; hCG: chorionic gonadotropin; CTx: chemotherapy.

A right orchidectomy was readily executed, and the histology revealed testicular involvement by mixed germ cell neoplasm associated with germ cell neoplasia in situ, with seminoma-type components, embryonal carcinoma type, and yolk sac type (Figure 4). At the time of the surgery, serum tumoral biomarkers such as alpha-fetoprotein (AFP -47.9 mg/dL (*N* 1-8 mg/mL)), chorionic gonadotropin (hCG -4.3 mUI/mL (*N* 0-4 mUI/L)), and lactate dehydrogenase (LDH -239 U/L (*N* 135-225 U/L)) were elevated (Figure 5).

The case was presented and discussed on a multidisciplinary team conference, and chemotherapy with four cycles of bleomycin, etoposide, and cisplatin (BEP regimen) was chosen based on lymph node spreading.

At the beginning of chemotherapy, about 2 months after surgery, tumoral markers reached a maximum peak. Then, in the following two months, they finally decreased to normal values and remained normal until the present day (Figure 5).

**Figure 6:**
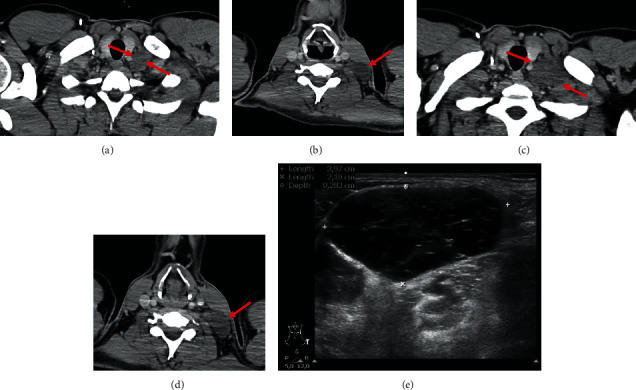
CT and ultrasound. Axial thoracic contrast-enhanced CT images at 2-month (a, b) and 5-month follow-up (c, d), showing progressive enlargement of left cervical lymph nodes with diffuse hypodensity (red arrows). (d) Ultrasound image of the same left cervical lymph node depicted on the CT images (a–d) at the microbiopsy, which showed mixed echogenicity, being primarily hypoechogenic with some echogenic septa.

**Figure 7:**
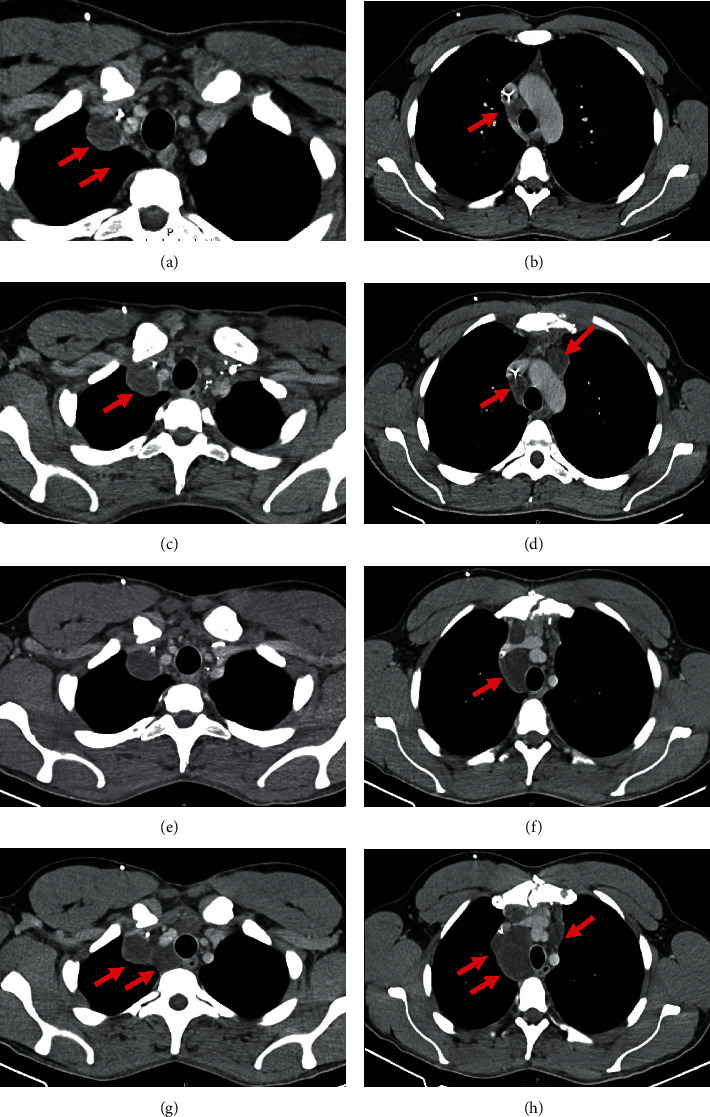
CT images. Axial thoracic contrast-enhanced CT images on two different mediastinal levels at 5-month (a, c), 7-month (c, d), 12-month (e, f), and 24-month (g, h) follow-up, depicting enlarging hypodense lymph nodes.

On the other hand, follow-up CT imaging, performed about every 2 to 3 months, demonstrated increased number and larger cervical and retroperitoneal lymph nodes. Furthermore, it showed new mediastinal lesions with similar appearance—diffuse hypodensity—suggesting cystic/necrotic nature (Figures 6 and 7).

**Figure 8:**
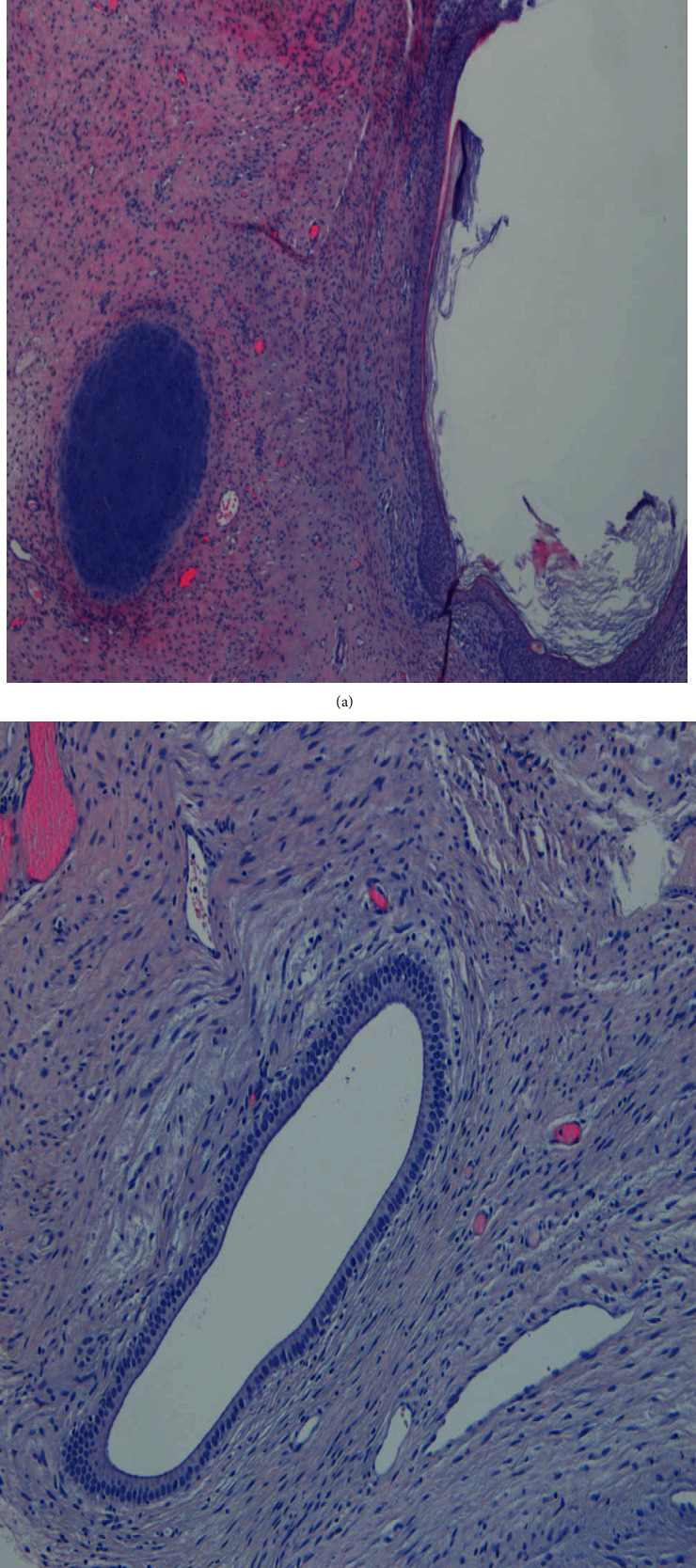
Lymph node metastases from germ neoplasia with characteristics of mature teratoma with components from the three germinal layers (a, b, H&E 100x).

Five months postsurgery, when the chemotherapy regimen was completed, a microbiopsy was performed on a left cervical lymphadenomegaly (Figure 8). Since this lesion's histology was compatible with teratoma, and there was normalization of tumoral biomarkers and tumor growth during and after systemic chemotherapy after a significant reduction in tumor burden, the diagnosis of growing teratoma syndrome was suggested.

On the following multidisciplinary team meeting, excision of the cervical and retroperitoneal secondary lesions was proposed and the patient underwent cervical and retroperitoneal lymphadenectomies without major complications.

At 24 months of surveillance, tumoral biomarkers remained normal, and there were not new cervical or retroperitoneal secondary lesions to report on imaging. However, the mediastinal lesions persisted and showed slow size progression over time, but considering the known risks associated with their anatomical location, an active surveillance approach was decided. At the moment, the patient is compliant with the follow-up protocol without major events or complications associated.

## 3. Discussion

Growing teratoma syndrome (GTS) was first described by Logothetis in 1982, as a rare entity among patients with nonseminomatous germ cell tumors [1, 2]. It is characterized by growing metastatic masses despite appropriate systemic chemotherapy and normal serum markers. This condition remains poorly known, with most of the reports consisting of case series with few patients [1–5].

According to Logothetis, the diagnostic criteria of GTS were (a) normalization of elevated serum AFP and hCG, (b) tumor growth during or after systemic chemotherapy, subsequently to a significant reduction in tumor burden or a disease-free interval, and (c) the exclusive histologicallypresence of mature teratoma in the resected specimen [6, 7].

GTS is now considered a condition related to both testicular and ovarian cancer (NSGCT), with a prevalence of about 1.9–7.6% in patients with testicular NSGCT [1, 7].

The most common site of involvement is the retroperitoneum, but it can be found in other locations such as the pelvis, chest, liver, or even in the cervical region as our case [3, 7].

GTS presentation is very variable regarding disease time course: during chemotherapy, after a large disease-free period, or after an initial reduction in size during chemotherapy, followed by regrowth [1, 7].

The etiology is still uncertain but there are two main hypotheses described in the literature based on the assumption that embryonal cells are pluripotent and that their origin results from differentiation of tumoral cells. One theory states that chemotherapy only cures the immature malignant cells but not the mature elements, which remain untreated and can transform into benign teratomatous elements. The other theory explains the transformation of a totipotent malignal germ cell into a benign mature teratoma by cell kinetics alterations stimulated by chemotherapy. There is still another lesser known hypothesis proposed by Hong et al., in which inherent and spontaneous differentiation of malignant cells into benign cells is pointed out as the cause of this pathology [4, 7–10].

Since this entity is rare, it is difficult to infer a statistically significant association between the primary pathology and the likelihood of GTS [1].

The most common risk factors include presence of mature teratoma in the primary tumor, incomplete resection after chemotherapy, no size reduction or even growth of metastatic lesions during chemotherapy, and presence of mature teratoma tissue in postchemotherapy residual masses [7, 11].

Among these mentioned risk factors, our patient presented with the two last ones.

However, according to recent studies, the only predictive factor associated with GTS syndrome seems to be cystic/necrotic lesions in the preoperative imaging as well as areas of low density on them at postchemotherapy CT scans compared to pretreatment images. Therefore, close imaging surveillance is crucial for early recognition of GST, particularly in patients with risk factors [1, 3, 11, 12, 13].

The residual masses of GTS do not demonstrate enhancement with 18-FDG PET (positron emission tomography) scans, which can be helpful in the differential diagnosis [14].

The chemoresistant nature of GTS makes additional cycles of chemotherapy ineffective; thus, early recognition is critical to prevent further systemic therapy and to allow for readily surgical planning. Surgical resection is currently the gold standard treatment for GTS. Despite the large tumor burden, complete resection is reported to be often curative. Failure to promptly intervene leads to death from either compression of vital organs and/or subsequent malignant transformation [6, 7].

Giant GTS is a variant of this syndrome, with extensive retroperitoneal disease, defined as lesions with larger than 10 cm. Circumferential involvement of major vascular structures is often seen. Surgery is usually technically challenging but should not be disregarded [1].

Although GTS generally has a good prognosis, regular follow-up is critical, as malignant degeneration could occur. Imaging findings and tumor markers can suggest the presence of malignant lesions, but surgical excision or sampling of the lesions is mandatory to confirm the diagnosis and other differential diagnosis as incomplete response to chemotherapy or recurrent malignancy [4].

## 4. Conclusion

GTS is rare condition related to both testicular and ovarian NSGCT with generally good prognosis, but close imaging surveillance is crucial for early recognition, particularly in patients with risk factors. Surgery is the gold standard treatment.
